# Ecdysis Triggering Hormone Signaling (ETH/ETHR-A) Is Required for the Larva-Larva Ecdysis in *Bactrocera dorsalis* (Diptera: Tephritidae)

**DOI:** 10.3389/fphys.2017.00587

**Published:** 2017-08-22

**Authors:** Yan Shi, Hong-Bo Jiang, Shun-Hua Gui, Xiao-Qiang Liu, Yu-Xia Pei, Li Xu, Guy Smagghe, Jin-Jun Wang

**Affiliations:** ^1^Key Laboratory of Entomology and Pest Control Engineering, College of Plant Protection, Southwest University Chongqing, China; ^2^Academy of Agricultural Sciences, Southwest University Chongqing, China; ^3^Department of Crop Protection, Faculty of Bioscience Engineering, Ghent University Ghent, Belgium

**Keywords:** *Bactrocera dorsalis*, ecdysis-triggering hormone, ecdysis, larva development, RNAi

## Abstract

Insects must undergo ecdysis for successful development and growth, and the ecdysis triggering hormone (ETH), released by the Inka cells, is a master hormone in this process. In this study, we determined the sequence of the ETH precursor and receptors in an agriculturally important pest insect, the oriental fruit fly *Bactrocera dorsalis* (Hendel). We identified two functionally distinct splice receptor isoforms: BdETH-R-A and BdETH-R-B, and when expressed in Chinese hamster ovary (CHO-WTA11) cells, they exhibited a high sensitivity to the two mature peptides BdETH1 and BdETH2. The *BdETH* transcript was detected in the tracheal tissue of the larvae. Inka cells were identified with immunohistochemical antibody staining against *Drosophila melanogaster* ETH1, and *in situ* hybridization with specific DNA probes. Selective RNA silencing of *BdETH* or *BdETH-R-A*, but not of *BdETH-R-B*, caused developmental failure at ecdysis. The dsRNA-treated larvae displayed tracheal defects and could not shed the old cuticle followed by death. Our results demonstrated that BdETH, via activation of BdETH-R-A but not ETH-R-B, plays an essential role in regulating the process of larva-larva ecdysis in *B. dorsalis*.

## Introduction

In insects, ecdysis is the process of shedding of the old cuticle, that is critical for their successful growth and development, and it is under control of multiple hormones (Gilbert et al., 2000; Truman and Riddiford, 2002; Arakane et al., 2008; Krüger et al., 2015; Mena et al., 2016). Ecdysis triggering hormone (ETH) is the key triggering factor in the neuropeptidergic cascade regulating ecdysis, and it was first discovered in *Manduca sexta* (Žitňan et al., 1996). During the pre-ecdysis and ecdysis phases, ETH acts directly on the central nervous system (CNS) to coordinate the muscle contractions (Roller et al., 2010). During the process of molting, a new and large tracheal system, which is filled with liquid, is generated around the smaller old tracheae (Snelling et al., 2011). The breakdown of the old trachea and the inflation of new tracheae are key events during ecdysis (Kim et al., 2015). Most lepidopterans produce two ETH peptides, PETH and ETH, whereas ETH1 and ETH2 peptides are found in other insect orders (Žitňan et al., 2002). ETH1 and ETH2 contain the conserved C-terminal sequence motif -KxxKxxPRx amide (Roller et al., 2010). Both of these ETHs are cleaved from a unique precursor gene, and are produced and secreted by specialized secretory cells, named Inka cells and these are located in the epitracheal glands (Kingan and Adams, 2000). The presence of the Inka cells, along with the ETH signaling, is highly conserved in diverse insect groups, suggesting that their functions are also conserved (Žitňan et al., 1996, 1999, 2003; Kingan et al., 1998; Park et al., 1999; Li and Adams, 2009; Roller et al., 2010; Bhagath et al., 2016). In *Drosophila melanogaster*, knocking out of the ETH gene resulted in lethal phenotypes that included failure of the respiratory system inflation and disability of the ecdysis behavioral sequence. This also led to lethality during the first instar larval ecdysis with a typical *buttoned-up* phenotype. This phenotype was originally described in ETH-null mutants characterized by double mouthparts, elimination of ecdysis behaviors, and failure to shed the old cuticle (Park et al., 2002; Cho et al., 2014). ETH appeared crucial for insect ecdysis (Park et al., 2002; Arakane et al., 2008; Lenaerts et al., 2017), and the ETH signaling pathway can be considered a potential target for insect control.

The ETH receptor is a typical G protein-coupled receptor, and two functionally distinct isoforms ETH-R-A and ETH-R-B are reported as being result of alternative splicing and have been observed in different insect species (Roller et al., 2010). The ligand sensitivity and specificity of the two receptor isoforms are different, and they are expressed in separated populations of central neurons, suggesting that they have distinctive roles in the ETH signaling pathway (Park et al., 2003; Diao et al., 2015). Silencing of the ETHR in different holometabolous species resulted in lethality at ecdysis (Park et al., 2002; Bai et al., 2011; Lenaerts et al., 2017).

The oriental fruit fly, *Bactrocera dorsalis* (Hendel), is one of the most destructive pests of agriculture. It attacks over 250 host plants worldwide and has become a great threat to fruit and vegetable industries because of its high reproductive capacity and invasiveness. Due to rapid development of insecticide resistance, the control of this pest has become difficult (Stephens et al., 2007). There are several reports about the neural and hormonal control of growth and development of *B. dorsalis* (Xu et al., 2015; Wu et al., 2016), but the exact mechanism of regulation remains unknown.

In this study, we cloned the cDNA sequences of ETH, ETH-R-A and ETH-R-B of *B. dorsalis*. We also investigated the expression of these genes in the different stages and tissues during the larval development. Then to confirm the real-time quantitative PCR (RT-qPCR) results, we performed immunohistochemistry and *in situ* hybridization also to locate the Inka cells. Finally, we used RNA interference (RNAi) assays by the feeding of dsRNA against *ETH, ETH-R-A* and *ETH-R-B* to the larval stages of *B. dorsalis* for studying the functions of these ETH signaling elements in insect growth and development, especially in the process of larva-larva ecdysis.

## Materials and methods

### Insects and reagents

Insects were reared as previously described (Shen et al., 2013). Briefly, *B. dorsalis* were reared in a laboratory at 27°C, relative humidity of 70%, and a day/night (14:10 h) photoperiod cycle. Adult females oviposited into pinpricked plastic tubes containing fresh orange juice and the eggs were collected. Newly hatched larvae and newly emerged adults were fed with different artificial diets as previously described (Wang et al., 2013).

### Molecular cloning

Based on the genome database of *B. dorsalis* (https://i5k.nal.usda.gov/Bactrocera_dorsalis), the *BdETH* and *BdETH-R* genes were identified by performing a TBLASTN search for the *ETH* and *ETH-R* homologs in *D. melanogaster* (Park et al., 2002, 2003). RNA was extracted from different developmental stages (eggs, larvae at the early stage of the 1st, 2nd, and 3rd instar; larvae at the late stage of the 1st, 2nd, and 3rd instar) and specific larval tissues including the central nervous system (CNS), fat body (FB), midgut (MG), Malpighian tubules (MT), integument (IN) and trachea (TR) using Trizol reagent (Invitrogen, Carlsbad, CA) according to the manufacturer's protocol. Single-strand cDNA was prepared using PrimeScript first-strand synthesis system (Takara, Dalian, China). Using high fidelity DNA polymerase PrimeSTAR (Takara), the full open reading frame (ORF) of BdETH and the transmembrane domains 4-7 of BdETH-R-A and BdETH-R-B were amplified. Primers were designed based on the genome data of *B. dorsalis* (Table S1) and PCR conditions were as follows: 98°C for 2 min, 95°C for 30 s, 60°C for 30 s and 72°C for 1 min with 35 cycles, and finally 72°C for 10 min. PCR products were purified and cloned into a pGEMT Easy vector (Promega, Beijing, China) and sequenced (BGI, Beijing, China).

### Sequence analysis

All sequences were aligned using ClustalX2 software (Larkin et al., 2007) with default settings. A neighbor-joining tree, gaps/missing date-pairwise deletion, was produced in MEGA 5.0 (Tamura et al., 2011) with 1,000 bootstrap replicates. Transmembrane helices were predicted using the TMHMM server (http://www.cbs.dtu.dk/services/TMHMM). The SignalP server (http://www.cbs.dtu.dk/services/SignalP) was used to predict signal peptide, and Weblogo (Crooks et al., 2004) to generated the sequence logos for the C-terminal motifs of ETH.

### Heterologous expression and functional assay

The ORFs for BdETH-R-A and BdETH-R-B were inserted into the expression vector pcDNA3.1 (+) with restriction enzymes (pcDNA3.1-*BdETH-R-A*: Kpn I and Xba I; pcDNA3.1-*BdETH-R-B*: Hind III and Not I). The correct clones of pcDNA3.1-BdETH-R-A and pcDNA3.1-BdETH-R-B were confirmed by sequencing (BGI). The Chinese Hamster Ovary (CHO-WTA11) cells, supplemented with aequorin and the Gα16 subunit, were employed for the heterologous expression. The methods for the transfection and the Ca^2+^ mobilization assay was performed as previously described (Jiang et al., 2013; Gui et al., 2017). All experiments were replicated three times.

### Real-time quantitative PCR (RT-qPCR)

A Stratagene Mx3000P system (Stratagene, La Jolla, CA) using iQ-SYBR Green Supermix (Bio-Rad, Hercules, CA) was used for RT-qPCR. The 10 μl of reaction system consisted of 5 μl of SYBR Green Supermix, 3.5 μl of nuclease free water, 0.5 μl of each primer (10 mM), and containing 0.5 μL of cDNA samples (~200 ng/μL). The PCR conditions were 95°C for 2 min, and 40 cycles of 95°C for 15 s and 60°C for 30 s, 95°C for 1 min. This was followed by melting curve analysis (60–95°C). Based on our previous evaluations (Shen et al., 2010), α-tubulin (GU269902) was used as a reference gene. To determine the amplification efficiencies, a standard curve was established for each primer pair with serial dilutions of cDNA (1/1, 1/5, 1/25, 1/125, and 1/625). Four biological replicates were performed for the samples collected from each developmental stage or tissue, and the data were analyzed with qBase software (Hellemans et al., 2007).

### Immunohistochemistry and *In situ* hybridization

An antibody against the ETH peptide of *D. melanogaster* (DmETH1, DSSPGFFLKITKNVPRLamide) (Park et al., 2002) was obtained from Dr. Yoonseong Park (Kansas State University, KS). The tracheae of *B. dorsalis* larvae were dissected in chilled 0.01 mol/L PBS, fixed for overnight in fresh 4% paraformaldehyde in PBS at 4°C and then rinsed three times (5 min each) in 0.01 mol/L PBS with 0.5% Triton X-100 (PBST). The immunohistochemistry stained tracheae with attached Inka cells were incubated with primary antisera for 2 days at 4°C at a 1:1,000 dilution of DmETH1 antiserum (Park et al., 2002). To reveal specific binding of the primary antibodies, we used Alexa 488-conjugated goat anti-rabbit IgG antibody (1:1000 dilution in PBST). Tissues were washed twice for 10 min with PBST and stained in 4′,6′-diamino-2-phenylindole (DAPI: 2 μg/ml) (Sigma, St Louis, MO) for 15 min. Labeled tissues were mounted on a clean slide with 100% glycerol. Images were captured with a confocal microscope (Zeiss LSM780, Zeiss, Jena, Germany).

*In situ* hybridization was conducted as previously described (Jiang et al., 2013; Gui et al., 2017). The ETH probes were prepared by the method of asymmetric PCR using a DIG Probe Synthesis Kit (Roche, Mannheim, Germany). Dissected tracheae were fixed overnight in 4% paraformaldehyde at 4°C, washed three times for 5 min each with PBST0.2 (PBS and 0.2% Triton-X-100), treated with 50 μg/ml proteinase K for 12 min, and the reaction was stopped with PBST0.2-glycine (2 mg glycine and 1 ml PBST0.2) for 5 min. Tracheae were refixed in 4% paraformaldehyde for 1 h, and hybridized with digoxin-labeled probes at 48°C for 24 h. After hybridization, tissues were washed with hybridization solution, blocked in 5% normal goat-serum for 30 min, and incubated with primary antibody (rabbit antidigoxin, 1:500; Bioss, Beijing, China) in PBST0.2 for 2 days at 4°C, and then followed by three washes of 5 min each with PBST0.2. Tissues were then incubated with secondary antibody (Dylight 488 labeled goat antirabbit IgG antibody) and mounted in DAPI for 15 min. Finally, images were captured as described above.

### RNA interference (RNAi)

To explore the functions of ETH signaling in *B. dorsalis*, an oral RNAi bioassay was performed. The most unique nucleotide regions of *BdETH, BdETH-R-A* and *BdETH-R-B* were selected to design the specific dsRNA sequences (Table S1), and *dsGFP* was used as a negative control. According to the manufacturer's instructions, these dsRNAs were synthesized with the Transcript Aid T7 High Yield Transcription Kit (Thermo Scientific, Vilnius, Lithuania). A self-designed setup was used to silence genes by dsRNA feeding. Briefly, the setup consisted of a sterilized Petri dish cover and 1.5 ml-Eppendorf tubes. In the cover, 20 μl of dsRNA (2,000 ng/μl) was added, and then newly hatched 1st instar larvae or newly molted 2nd instar larvae were transferred into the tube cover and maintained for 3 days. We performed three biological repeats and each consisted of 30 larvae from each group (treatment and control). The insects were provided with a small amount of artificial diet added in the tube cover, and the dsRNA was replaced every 24 h. At 48 h after the feeding, larvae were collected and RT-qPCR was performed to calculate the RNAi efficiency. The mortality percentages were observed daily for 3 days.

### Phenotype observation

The 1st, 2nd and 3rd instar larvae of *B. dorsalis* were distinguished morphologically as described by Shi et al. (2017). The larvae exhibiting defects after dsRNA treatment were positioned in a drop of tap water on a glass slide. Shapes of tracheae and sclerotized structures were observed and photographed with a Leica M205A stereomicroscope (Leica Microsystems, Wetzlar, Germany) to illustrate the morphological characters of the defects.

### Statistics analysis

All statistical analyses were performed in SPSS 20.0. For the gene expression profiles, one-way ANOVA followed by Tukey test was applied to test for significant differences among different developmental stages or tissues. A student's *t*-test was used to determine the significance of differences between the treatment and control in the dsRNA feeding assay. Means ± SE (standard error) were determined based on three biological replications.

## Results

### Sequences analysis and a phylogenetic tree construction

**Figure 1:**
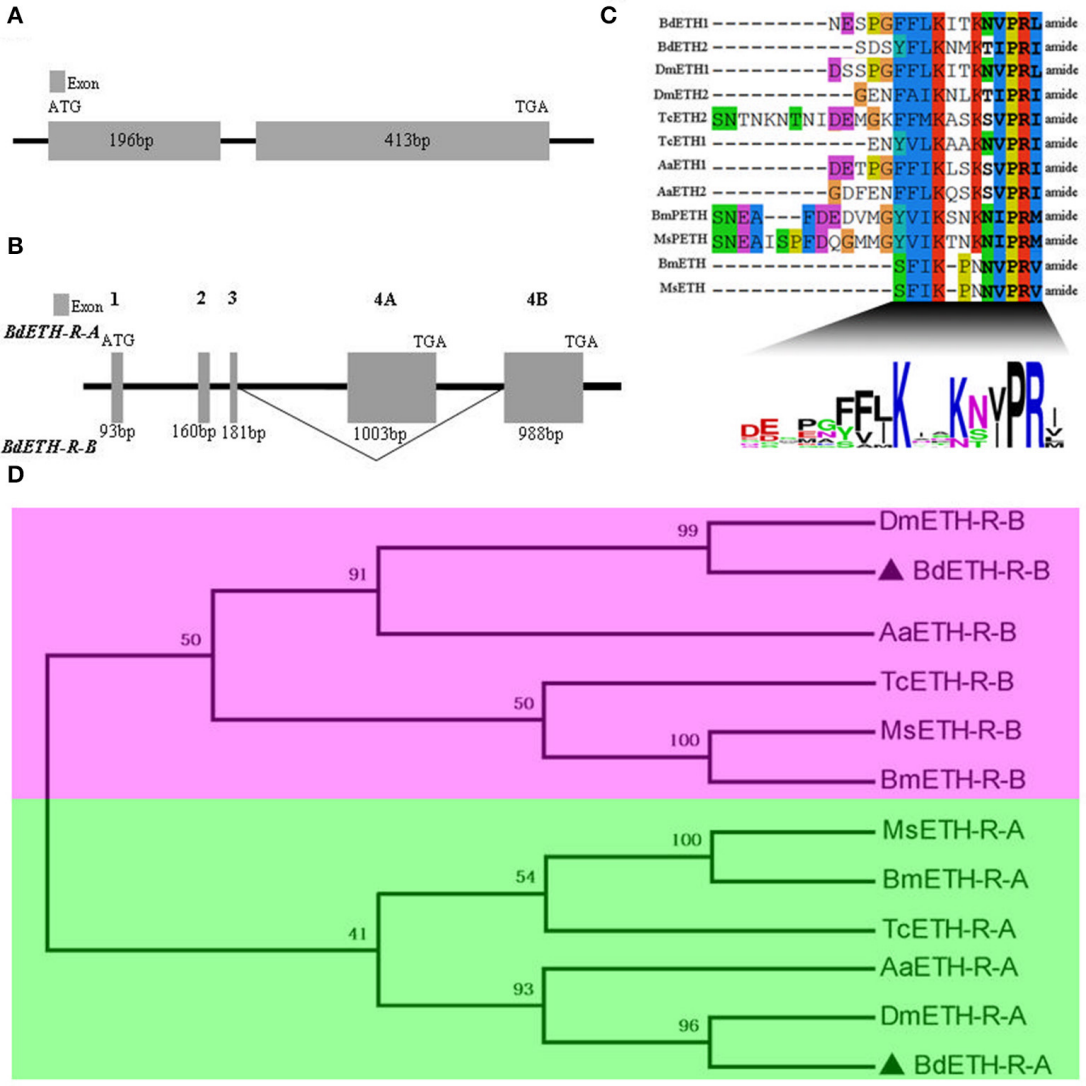
Gene structure of BdETH, BdETH-R-A, BdETH-R-B and phylogenetic relationships of ETHRs. **(A)** Gene structure of *B. dorsalis* ETH, predicted from the genome. **(B)** Organization of the predicted *B. dorsalis* ETH-R. The BdETH-R of *B. dorsalis* encodes two receptor isoforms resulting from alternative splicing of two mutually exclusive exons 4A and 4B. **(C)** An alignment showing consensus sequence of *B. dorsalis* and related insects putative mature peptides. The calculated consensus logo is shown at the bottom. **(D)** Phylogenetic relationships of alternatively spliced exons encoding isoforms A and B regions of ETH receptor and related GPCR (transmembrane domains 4–7). Bd, *Bactrocera dorsalis;* Dm, *Drosophila melanogaster;* Bm, *Bombyx mori;* Aa, *Aedes aegypti;* Ms, *Manduca sexta;* Tc, *Tribolium castaneum*.

The BdETH gene structure is shown in Figure 1A. It consists of two exons, which are 196 and 413 bp in length. The ORF encodes a protein of 203 amino acid. The two putative peptides BdETH1 and BdETH2 are divided by flanking dibasic cleavage sites (RR) (Figure S1). In addition, an amidated C-terminus was identified for each mature peptide by a canonical amidation site at the C-terminal end. Both of the mature peptides contain the signature motif KxxKxxPRxamide (“x” is a variable residue and “amide” represents the amidated C-terminus), and this typical motif is conserved across different insect species through an alignment (Figure 1C). The insect ETH mature peptide sequences used in this study are listed in Table S2.

**Figure 2:**
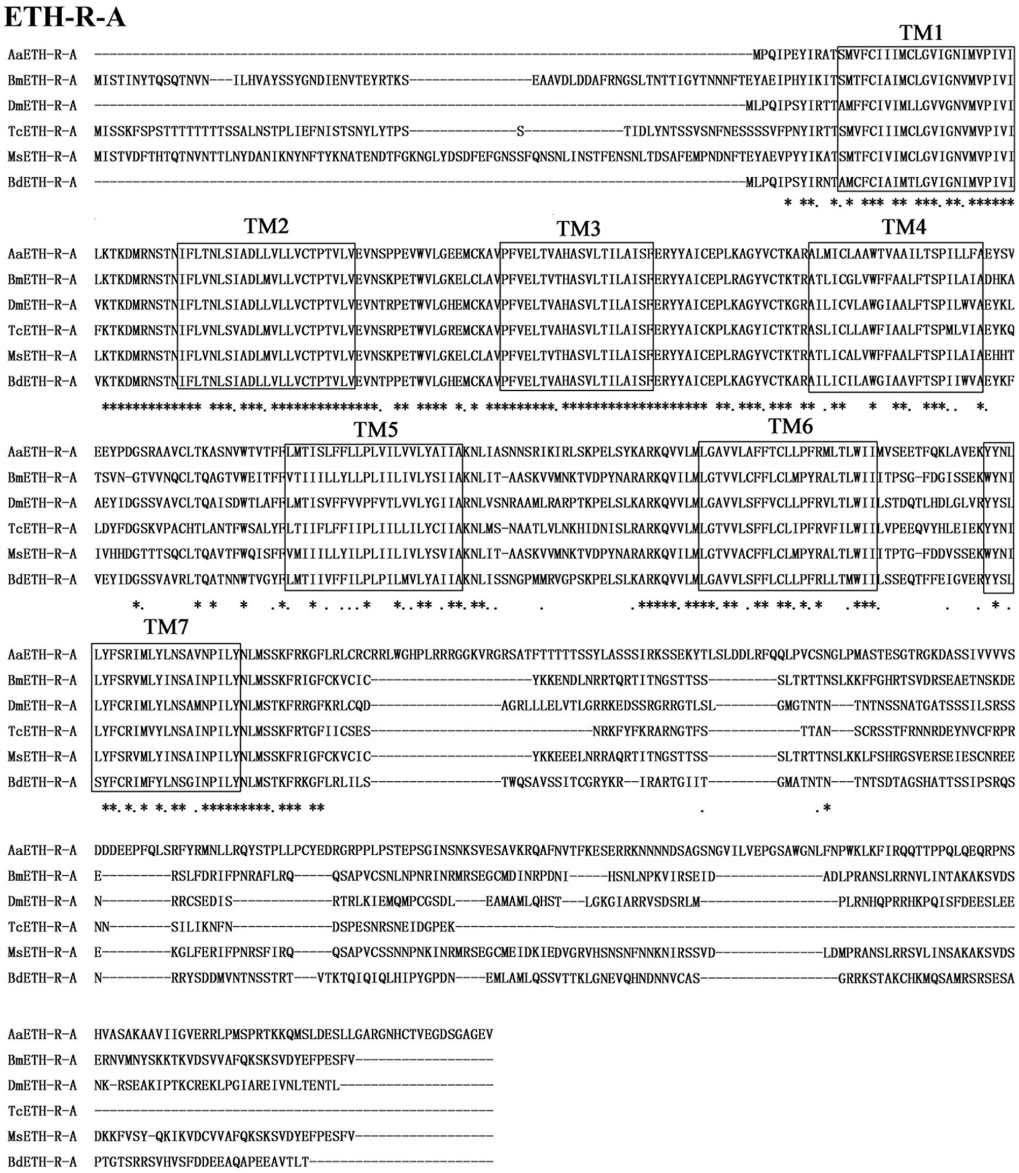
Amino acid sequence alignment of the BdETH-R-A with other related GPCRs. Conservative AA are indicated by asterisks. Seven transmembrane domains are highlighted in black box (TM1-TM7).

The BdETH-R-A and BdETH-R-B, consisting of four exons, are typical GPCRs with seven transmembrane domains (Figures 1B, 2 and Figure S2). Using the TMHMM server, we predicted the transmembrane domains 1-7 of BdETH-R-A and BdETH-R-B (Figure S3B), and domains 4–7 with the splicing sites were used for phylogenetic analysis. The phylogenetic analysis indicated that the two receptor isoforms are separated into two groups, and that each is closely related to ETH-R-A and ETH-R-B from other Diptera, such as *D. melanogaster* and *Aedes aegypti* (Figure 1D).

### Functional assay

**Figure 3:**
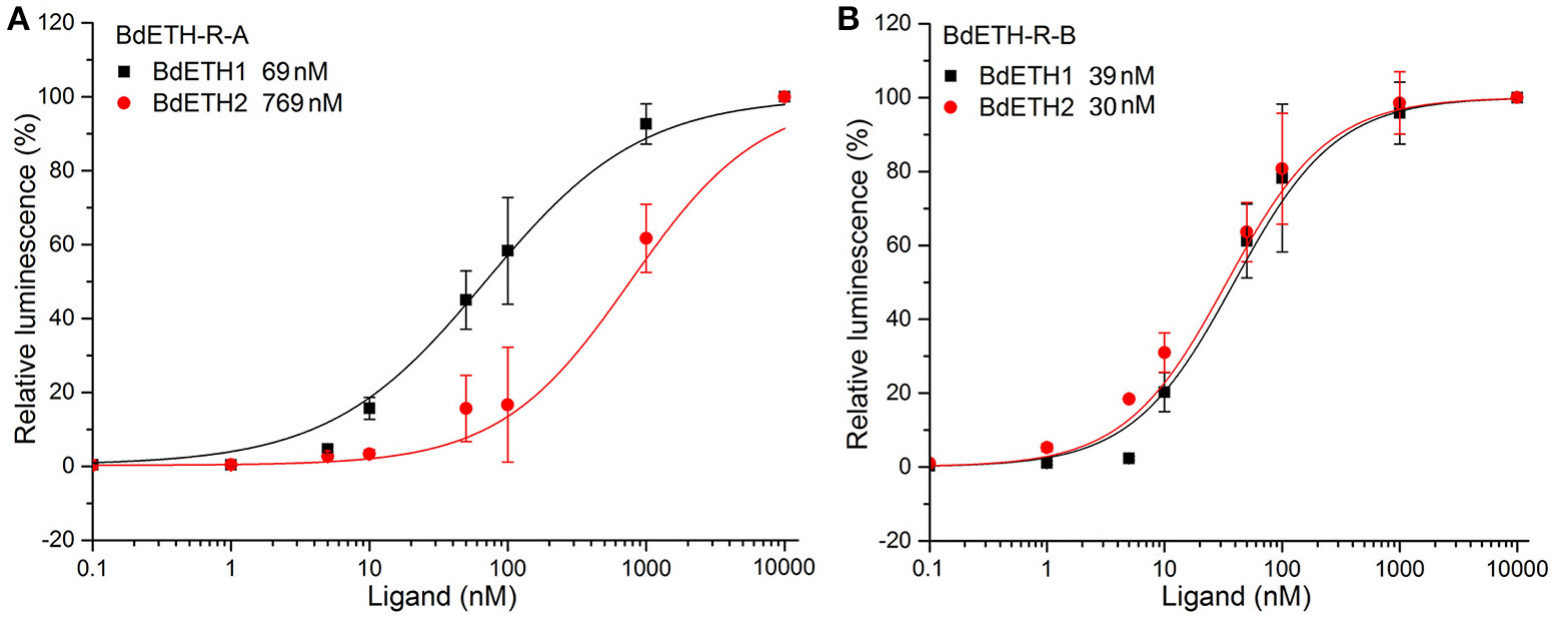
Ligand sensitivity of BdETH-R-A and BdETH-R-B expressed heterologously in CHO-WTA11 cells. **(A)** Concentration-response relationships for peptides (BdETH1 and BdETH2) tested on BdETH-R-A. **(B)** Concentration-response relationships for peptides (BdETH1 and BdETH2) tested on BdETH-R-B. Insets in each plot show EC_50_ values for each ligand.

To confirm that BdETH-R-A and BdETH-R-B are functional, a calcium reporter assay was performed with the two ETH peptides of *B. dorsalis* (Figure 3). Figure 3 shows that BdETH1 and BdETH2 both activated the BdETH-R-A and BdETH-R-B, expressed in CHO cells, and this in a concentration-dependent manner. The BdETH-R-A isoform was more sensitive to BdETH1 peptide (EC_50_ = 69 ± 31 nM) compared to BdETH2 (EC_50_ = 769 ± 182 nM), while BdETH1 (EC_50_ = 39 ± 16 nM) and BdETH2 (EC_50_ = 30 ± 11 nM) activated the BdETH-R-B to a similar extend.

### Transcriptional expression patterns

**Figure 4:**
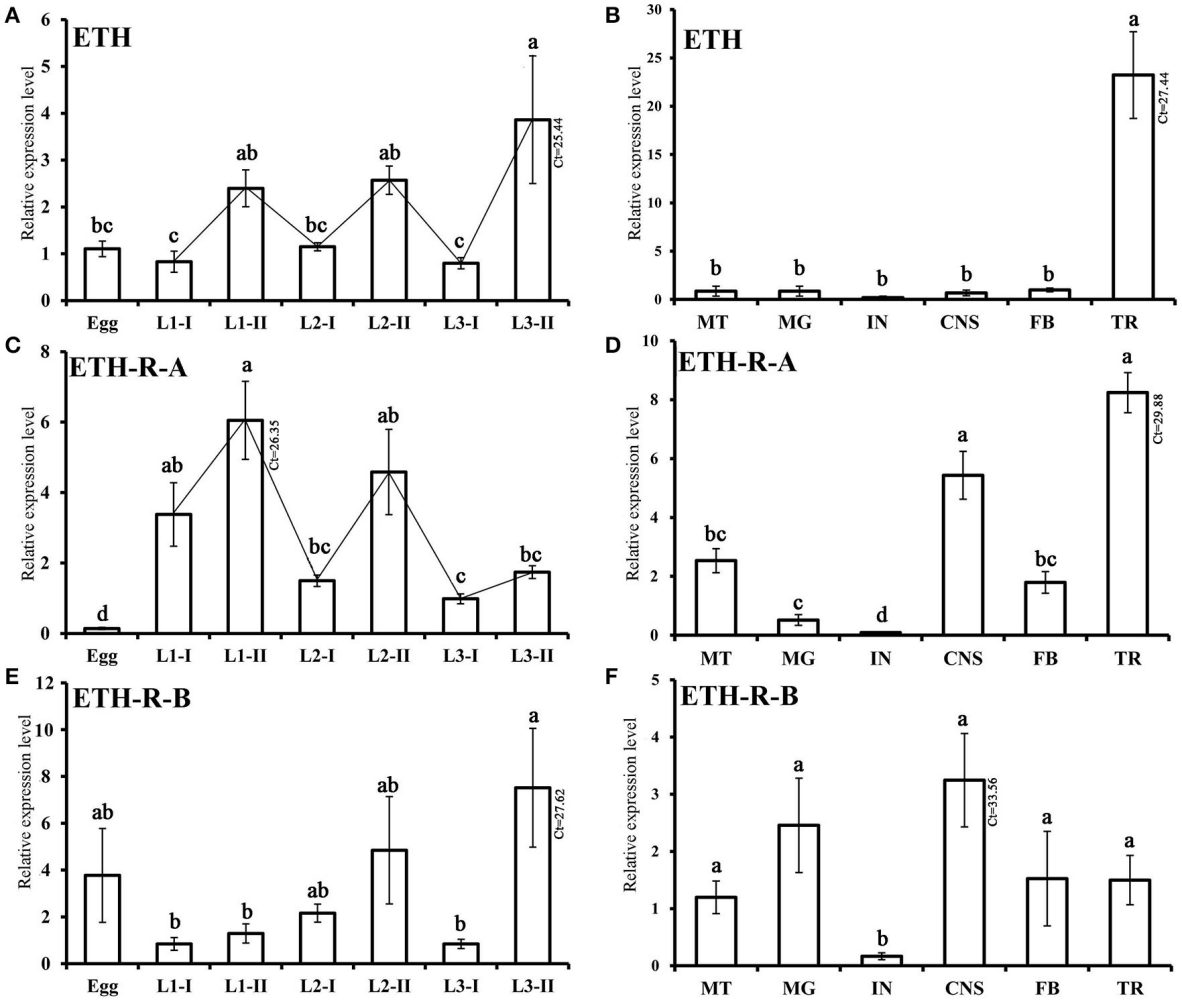
Relative transcripts of BdETH detected by RT-qPCR. Data are mean ± S.D. **(A,C,E)** Relative expression levels of *BdETH, BdETH-R-A* and *BdETH-R-B* in different developmental stages. Abbreviations used on X-axis: L1-I, L2-I, L3-I (larvae at early stage of the 1st, 2nd, 3rd instar), L1-II, L2-II, L3-II (larvae at the late stage of the 1st, 2nd, 3rd instar). **(B,D,F)** RT-qPCR determination of expression levels of *BdETH, BdETH-R-A* and *BdETH-R-B* in different tissues of larvae. Abbreviations used on X-axis: MG (midgut), CNS (central nervous system), FB (fat body), TR (trachea), MT (Malpighian tubules), IN (integument), Ct (cycle threshold). Different letters on the bars of the histogram indicate statistical differences based on the one-way ANOVA followed by Tukey's honest significant difference (HSD) multiple comparison test (*P* < 0.05).

The temporal and tissue-specific transcript profiles of *BdETH, BdETH-R-A* and *BdETH-R-B* were determined using RT-qPCR. *BdETH* was expressed in all developmental stages tested, but it was typical that for each instar that it showed a low expression at the early stage and a high expression at the late stage. This made its expression resemble a zigzag pattern (Figure 4A). *BdETH-R-A* showed an expression pattern matching with that of *BdETH* (Figures 4A,C). In contrast, the pattern of *BdETH-R-B* was different with a low to moderate expression in the 1st and 2nd in stars and the highest expression was seen at the late stage of the 3rd instar.

Tissue-specific expression levels of *BdETH, BdETH-R-A* and *BdETH-R-B* in 3rd instar larvae are shown in Figure 4B,D,F. For all three transcripts, low levels were found in the integument (IN). The highest levels of *BdETH* were recorded in the trachea (TR). *BdETH-R-A* was most expressed in the tracheae and CNS followed by the Malpighian tubes and fat body, while the profile of *BdETH-R-B* was different with an even and low expression in the CNS, midgut, Malpighian tubules, fat body and tracheae.

### Localization of ETH

**Figure 5:**
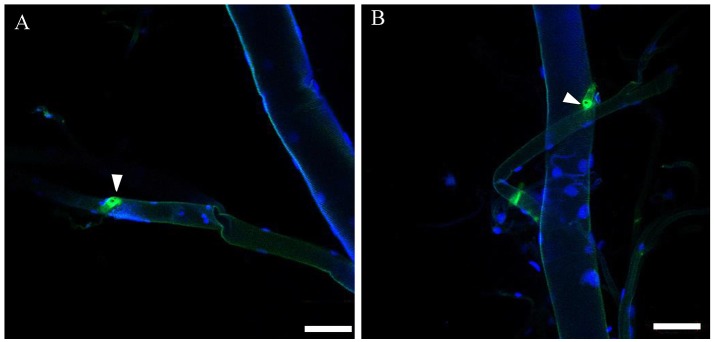
Localization of ETH Inka cells in trachea of *B. dorsalis* larvae. White triangles indicate Inka cells stained using immunohistochemistry and *in situ* hybridization. **(A)** Tracheae were dissected from larvae stained with an antiserum to DmETH1. **(B)** Tracheae of larvae stained with *in situ* DNA probe against *BdETH* mRNA. Scale bar, 100 μm.

By immunohistochemistry (IHC) using a rabbit antibody against the *D. melanogaster* ETH peptide, we localized the Inka cells that produce ETH in the tracheae of *B. dorsalis* at protein level (Figure 5A). To confirm the results from antibody staining, we used fluorescent *in situ* hybridization to localize the mRNA expression of *BdETH* by digoxin-labeled DNA probes. Similar to the IHC, strong signals were detected in the Inka cells at each branch point of the transverse connectives position of the tracheae in *B. dorsalis* (Figure 5B). The Inka cells occurred along each of the two dorsal tracheal trunks at the branch points of the transverse connectives (Figure 5). Negative controls for immunohistochemistry and fluorescent *in situ* hybridization (with a probe produced by the sense primer) were also carried out and no signal was detected (Figure S4).

### Observation of the phenotype upon RNAi

**Figure 6:**
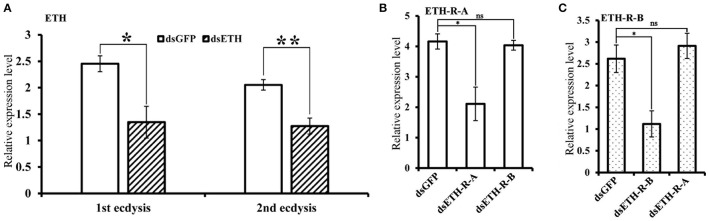
Silencing efficiency of genes of the ETH signaling (ETH/ETH-R) in *B. dorsali*s larvae. **(A)** The silencing efficiency of RNAi *BdETH* in 1st ecdysis and 2nd ecdysis. **(B)** The expression of *BdETH-R-B* when *BdETH-R-A* was knocked down in 1st ecdysis. **(C)** The expression of *BdETH-R-A* when *BdETH-R-B* was knocked down in 1st ecdysis. Data are means ± SD. Significant differences was determined using Student's *t*-test (^*^P < 0.05; ^**^P < 0.01). Abbreviation: dsGFP, double stranded green fluorescent protein; dsETH, double stranded ecdysis triggering hormone.

**Figure 7:**
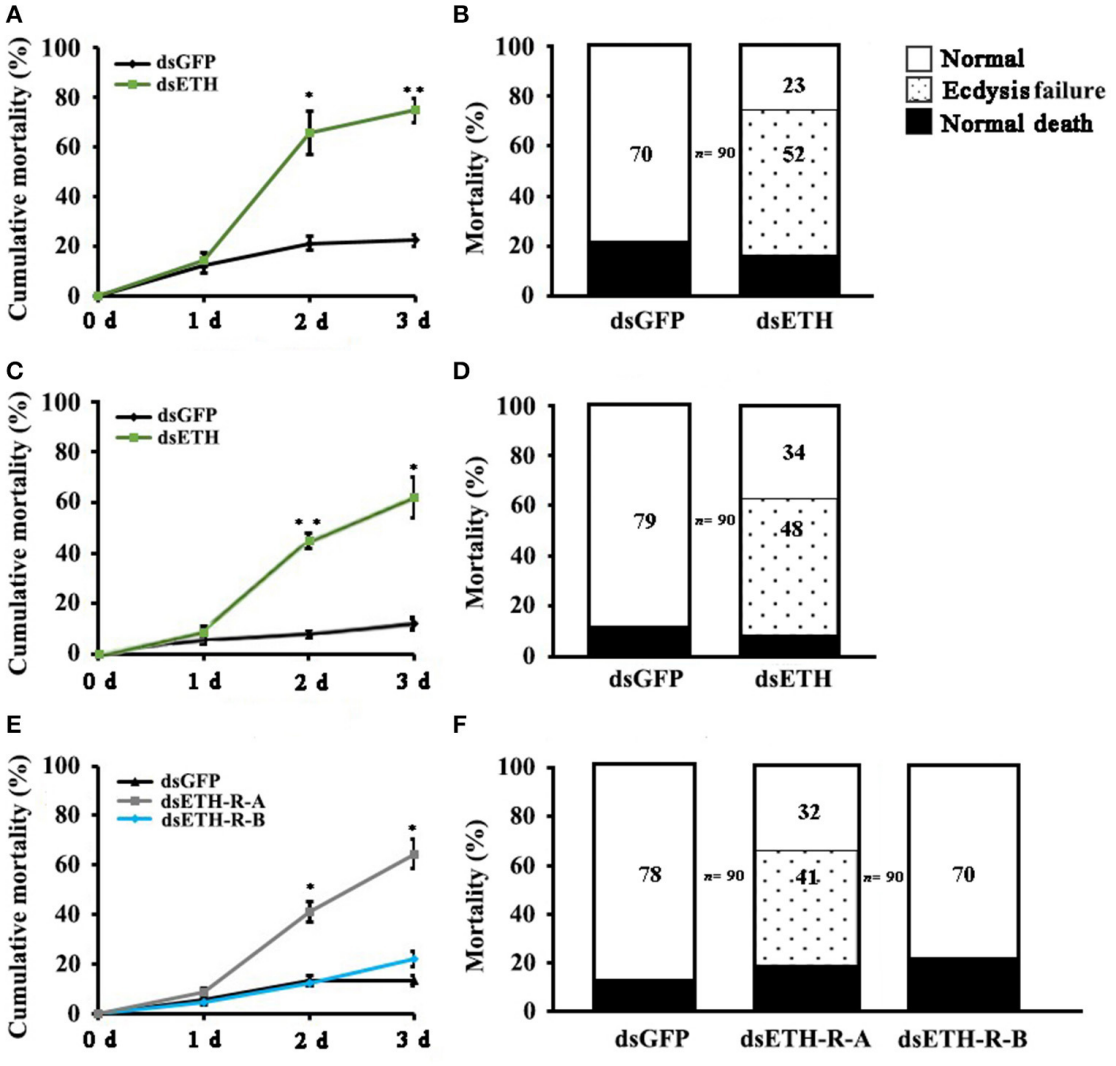
Impairment of ETH, ETH-R-A and ETH-R-B showed ecdysis failure. Percentages of defective larvae are displayed. Ecdysis failure, including tracheal defects and *buttoned-up* phenotype, eventually led to larval death. **(A)** Cumulative mortality of 1st instar larvae of *B. dorsalis* in the treatment and control after feeding dsRNA for 1, 2, and 3 days. **(B)** The 1st instar larvae treated with RNAi were scored for different phenotypes. **(C)** Cumulative mortality of 2nd instar larvae of *B. dorsalis* in the treatment and control after feeding dsRNA for 1, 2, and 3 days. **(D)** The 2nd instar larvae of RNAi scored for percent of different defects. **(E)** Cumulative mortality of 1st instar larvae of *B. dorsalis* in the treatment and control after feeding dsRNA for 1, 2, and 3 days. **(F)** The 1st instar larvae of RNAi were scored for presence of different phenotypes. Significance was determined using Student's *t*-test (^*^P < 0.05; ^**^P < 0.01).

**Figure 8:**
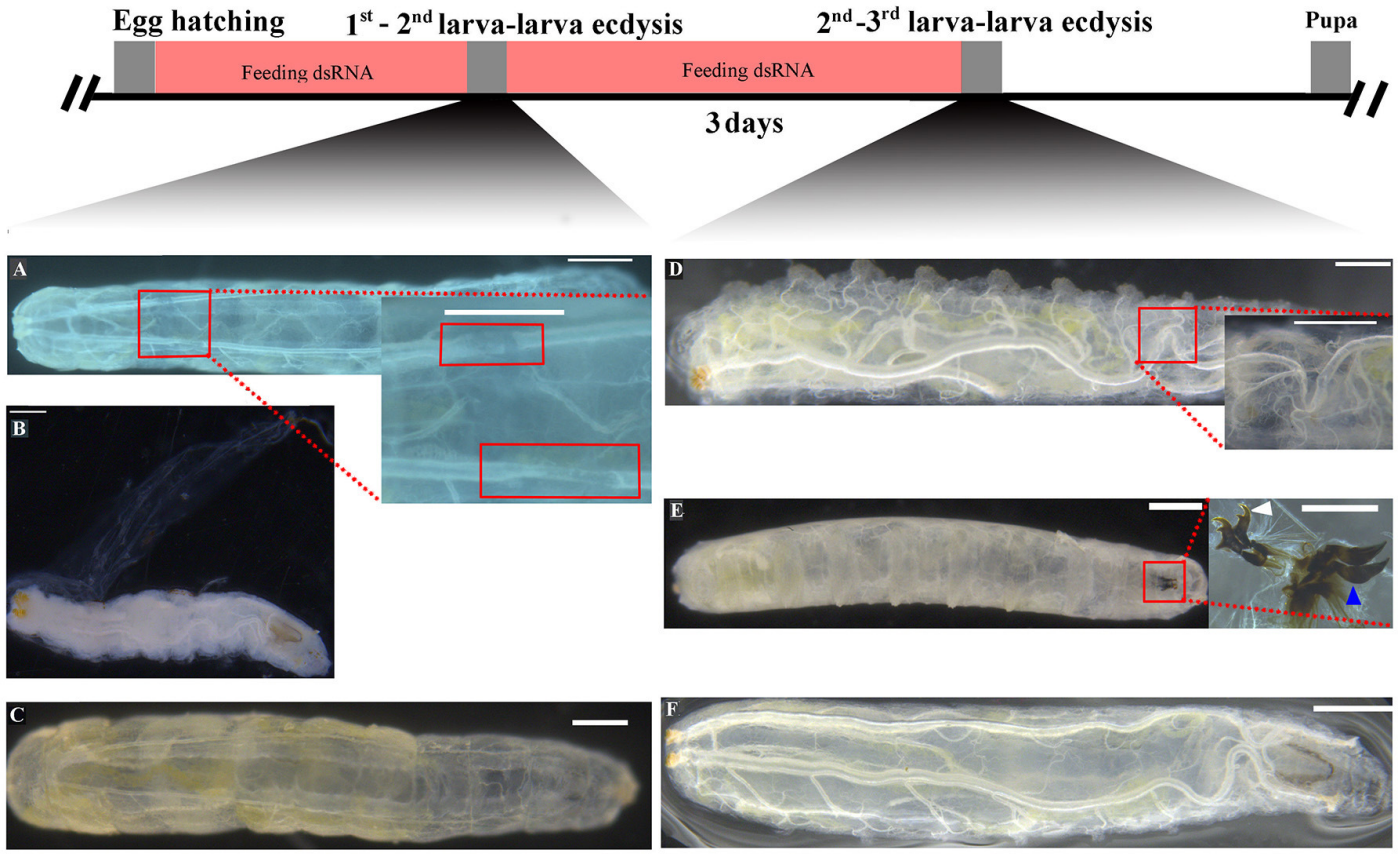
Knockdown of *BdETH* caused tracheal defects and *buttoned-up* phenotype. **(A)** New tracheae in 1st instar larvae have breaks. **(B)** Ecdysis failure and *buttoned-up* phenotype in the 1st - 2nd instar transition; ecdysis failure and death is indicated by partially shed old cuticle. **(D)** New tracheae in 2nd instar larvae have breaks. **(E)** Failure to shed old mouthparts indicated by double mouth hooks; white arrow indicates the mouth hook of a 2nd instar larvae; blue arrow indicates the mouth hook of a 3rd instar larvae. **(C,F)** Normally developed control larvae can ecdyse to the next instar. Scale bar, 200 μm. Inset picture scale bar, 100 μm.

The effects of RNAi-mediated knockdown of the *BdETH* expression in the 1st instar and 2nd instar larvae were observed. The knockdown efficiency in the 1st instar larvae was 49% at 48 h post-feeding of dsRNA (Figure 6A). Fresh dsRNA was added every 24 h, and the dead insects were recorded and removed. In the RNAi-1st instar larvae, the cumulative mortality was 74% after 72 h as compared to 22% in the controls (Figure 7A). The dsETH-treated larvae showed symptoms of disrupted respiratory dynamics and behavioral deficits, and finally these insects died after failure of ecdysis. A total of 41 out of 52 larvae died from tracheal defects such as incomplete shedding of the old tracheal lining and new tracheae with breaks (Figures 7B, 8A), and 11 of the 52 larvae died with an inhibition of ecdysis that is also known as the *buttoned-up* phenotype (Figures 7B, 8B).

In the RNAi bioassay with the early 2nd instar larvae, the knockdown efficiency was 41% at 48 h post-feeding of dsRNA (Figure 6A). The cumulative mortality of the dsRNA treatment was 62% after 72 h compared to 12% in the control (Figure 7C). Defective phenotypes were observed after *BdETH* was knocked down. A total of 48 larvae died from ecdysis failure. Of the 48 larvae, 39 had tracheal defects (Figure 8D). Nine larvae failed to shed their old mouthparts and had the *buttoned-up* phenotype (Figures 7D, 8E).

To characterize the ETH-R function, we also performed RNAi with specific sequences of *BdETH-R-A* and *BdETH-R-B* (Figure S3). During the 1st instar development, a significant reduction of *BdETH-R-A* and *BdETH-R-B* levels was seen and there were no off-target effects. A respective reduction of 51 and 52% in relative mRNA levels of *BdETH-R-A* and *BdETH-R-B* was measured at 48 h after the consumption of specific dsRNA (Figure 6B). When *BdETH-R-A* was knocked down, the cumulative mortality of the dsRNA treatment was 62% after 72 h compared to 13% in the control. The phenotype for the dsRNA-treated 1st instar larvae was similar to the treatment with dsETH in both 1st instar and 2nd instar (Figures 8A,B,E,F). At the expected time of ecdysis, the ds*BdETH-R-A*-treated larvae died of tracheal defects and *buttoned-up* phenotypes (Figures 7A–D). In great contrast, when *BdETH-R-B* was knocked down, the phenotypes of a larval ecdysis deficit were not obvious and nearly all, 78% (*n* = 90), of the 1^st^ instar larvae developed successfully into 2nd instar larvae (Figures 7E,F).

## Discussion

The *BdETH* precursor sequence was cloned from *B. dorsalis*. Two putative ETH peptides possessed the general motif sequence KxxKxxPRx at the C-terminal, found to be the ETH peptide motif in numerous insect species (Roller et al., 2010). The authenticity of the cloned ETH precursor sequence was substantiated by the ETH peptide motif. Each ETH peptide of *B. dorsalis* is highly conserved compared to the two ETH peptides of *D. melanogaster* (Figure 1C). The high degree of conservation in mature ETH peptides implies that the functions of *BdETH* might be similar to those observed in *D. melanogaster*.

On the ETH-Rs in *B. dorsalis*, our data showed that two distinct isoforms, *BdETH-R-A* and *BdETH-R-B*, are encoded by a single gene via alternative splicing of two 3′-exons (Figure 1B). These exons encode the protein sequence from the 4th transmembrane region to the C-terminus (Figure S3). The activation of BdETH-R-A and BdETH-R-B expressed in CHO-WTA11 cells mobilizes intracellular calcium. This finding is consistent with reports of ETHRs in *Drosophila* (Park et al., 2003; Kim et al., 2006). The concentration-response curves of *BdETH-R-A* and *BdETH-R-B* show that both are sensitive for the two mature ETH peptides of *B. dorsalis* at physiologically relevant concentrations (Figure 3). Based on median effective concentrations (EC_50_), there was one difference that the BdETH2 peptide was ~10-fold less active on the BdETH-R-A than BdETH1, but for BdETH-R-B, the two peptides could activate the receptor with similar EC_50_ values. Using immunohistochemistry, Inka cells have been located in representative species of the major insect orders. Inka cells are small, but numerous, and are scattered throughout the tracheal tissue (Žitňan et al., 2003; Roller et al., 2010). In this study, *BdETH* expression and localization were investigated using different methods, including RT-qPCR, immunohistochemistry and *in situ* hybridization. During the different developmental stages, *BdETH* mRNA reached high levels prior to each molt (Figure 4A). In different larval tissues, expression of *BdETH* precursor mRNA was high and nearly exclusive in the tracheal tissue. These data agree with the *BdETH* expression pattern in *D. melanogaster*, with a restricted expression in the tracheal tissue (Park et al., 2002), as reported in FlyAtlas (http://flyatlas.org/tissues.cgi). This indicates that *BdETH* may have the same function as in *D. melanogaster*. To further confirm the RT-qPCR results of the expression in different larval tissues, we performed *in situ* hybridization and immunohistochemistry. In larvae, Inka cells were observed along each of the two dorsal tracheal trunks at the branch points of transverse connectives (Figure 5), and also here our results of immunohistochemistry and *in situ* hybridization were consistent with *D. melanogaster* (Park et al., 2002; Cho et al., 2014). Thus, we believe that also in *B. dorsalis* the Inka cells can be considered are the source of ETH in the larval stages. Deletion of the ETH in *Drosophila* resulted in the inability of the respiratory system to inflate at the proper time (Park et al., 2002). In order to determine the function of ETH/ETH-R signaling in *B. dorsali*s, we downregulated the expression of ETH precursor transcript by RNAi and this resulted in the typical *buttoned-up* phenotype and tracheal defects in the 1st and 2nd instar larvae (Figure 8). In all cases, the *buttoned-up* phenotype resulted in ecdysis failure and the tracheal defects compromised the normal respiratory functions. The ecdysis failure contributed to the lethal phenotypes as affected larvae died soon afterwards. However, our results differed slightly in the cumulative mortality and the proportion of *buttoned-up* phenotypes compared to Park et al. (2002), who used null-mutants technology. We believe that this discrepancy may be a result of the lower percentages in gene silencing efficiency that were 50–60% in our RNAi bioassays. Consequently, some larvae in our RNAi bioassays succeeded to develop successfully into the next instar (Figures 7B,D). Interesting in our study was that the expression pattern of *BdETH* over the different developmental stages was similar to that of *BdETH-R-A*, but not to *BdETH-R-B* (Figures 4A,C,E). This suggests that the two receptor isoforms may have different roles in ETH signaling. Indeed, in *Drosophila, in situ* hybridization has shown that ETH-R-A and ETH-R-B are expressed in distinct populations of neurons (Kim et al., 2006). And also in the red flour beetle, global RNAi knockdown of the ETH receptor isoform A produced ecdysis deficits (Arakane et al., 2008). ETH-R-A expressing neurons are required for ecdysis throughout development, whereas ETH-R-B expressing neurons are required only after the larval stage (Diao et al., 2015). Our data together with these in *D. melanogaster* and *T. castaneum* strongly suggest that ETH-R-A and ETH-R-B have distinct functional roles and that their contribution to ecdysis is differentially dependent on developmental stage. In our RNAi bioassays, we followed *BdETH-R-A* and *BdETH-R-B* and found that 1st instar larvae treated with *BdETH*-dsRNA or *BdETH-R-A-*dsRNA have similar phenotypes, while the 1st instar larvae treated with GFP-dsRNA or *BdETH-R-B-*dsRNA had no tracheal defects and ecdysis failure phenotypes (Figures 7E,F). In addition, with the gene-silencing of *BdETH-R-A* and *BdETH-R-B*, also the expression of the other isoform was not affected and this was so for the two receptors, which confirms that the other isoform is not up-regulated to compensate the function of the silenced isoform (Figures 6B,C). Taken all together, our data suggest that the *BdETH* signaling is essential for larval ecdysis in *B. dorsalis*, and that *BdETH* appears to activate a myriad of downstream signaling pathways via activation of *BdETH-R-A* expression in the CNS. Overall, ETH-R-A expressing neurons are required for successful ecdysis throughout development of *B. dorsalis*.

In summary, we provide evidence that interfering with ETH signaling (*BdETH*/*BdETH-R-A*) caused tracheal defects and *buttoned-up* phenotypes during larva-larva ecdysis of *B. dorsalis*. After knockdown of *BdETH* or *BdETH-R-A*, the larvae had similar phenotypes in ecdysis and all affected larvae died. This study clarifies the functions of the ETH signaling pathway and suggests that the ETH/ETH-R-A can be an effective target for the control of pest insects such as *B. dorsalis*.

## Author contributions

HJ, GS, and JW designed research. YS performed all of the experiments with the help of SG, XL, YP, LX. GS and JW provided the materials. YS and GS analyzed data. YS, HJ, GS and JW wrote the paper.

### Conflict of interest statement

The authors declare that the research was conducted in the absence of any commercial or financial relationships that could be construed as a potential conflict of interest.
