# Supplementary material for: Ecdysis Triggering Hormone Signaling (ETH/ETHR-A) Is Required for the Larva-Larva Ecdysis in Bactrocera dorsalis (Diptera: Tephritidae)
Source: Front Physiol. 2017 Aug 22;8:587. doi: 10.3389/fphys.2017.00587 (PMC5572281; doi:10.3389/fphys.2017.00587)
Supplement: Supplementary file 1 [file Presentation1.PDF]

Supplementary Material

Ecdysis Triggering Hormone is required for the larva-larva Ecdysis in *Bactrocera dorsalis* (Diptera: Tephritidae)

Yan Shi[†1,2], Hong-Bo Jiang[†1,2], Shun-Hua Gui[1,2], Xiao-Qiang Liu[1,2], Yu-Xia Pei[1,2], Li Xu[1,2], Guy Smagghe[1,2,3] and Jin-Jun Wang[1,2*]

*** Correspondence:**

Jin-Jun Wang

wangjinjun@swu.edu.cn;

jjwang7008@yahoo.com

Guy Smagghe

Guy.smagghe@ugent.be

>BdETH Precursor

MHSSSLLTFGVLLLLTTLANHCQGNESPGFFLKITKNVPRL***GRR***SDSYFL
KNMKTIPRI***GRR***GDGELDVALLPSLSKRMLMNPAEAAAAAEREYSLVQPV
TSNTLIELLNKNAIAADNIKFVHWKDFDRALQRDTELYGKLISLGRKPDQ
RLKADLNIDMNSGFTPLVSSSNSNDYIYYNKDVDEMYAPKYGGDFMRYNQ
LD-

Figure S1. Deduced amino acid sequences of *Bactrocera dorsalis* ETH. Consecutively, coding sequences for signal peptide (underlined sequences) and predicted ETH-peptides (sequences in green and red colour). Predicted amidation signal with dibasic cleavage sites (sequences in bold and italic).

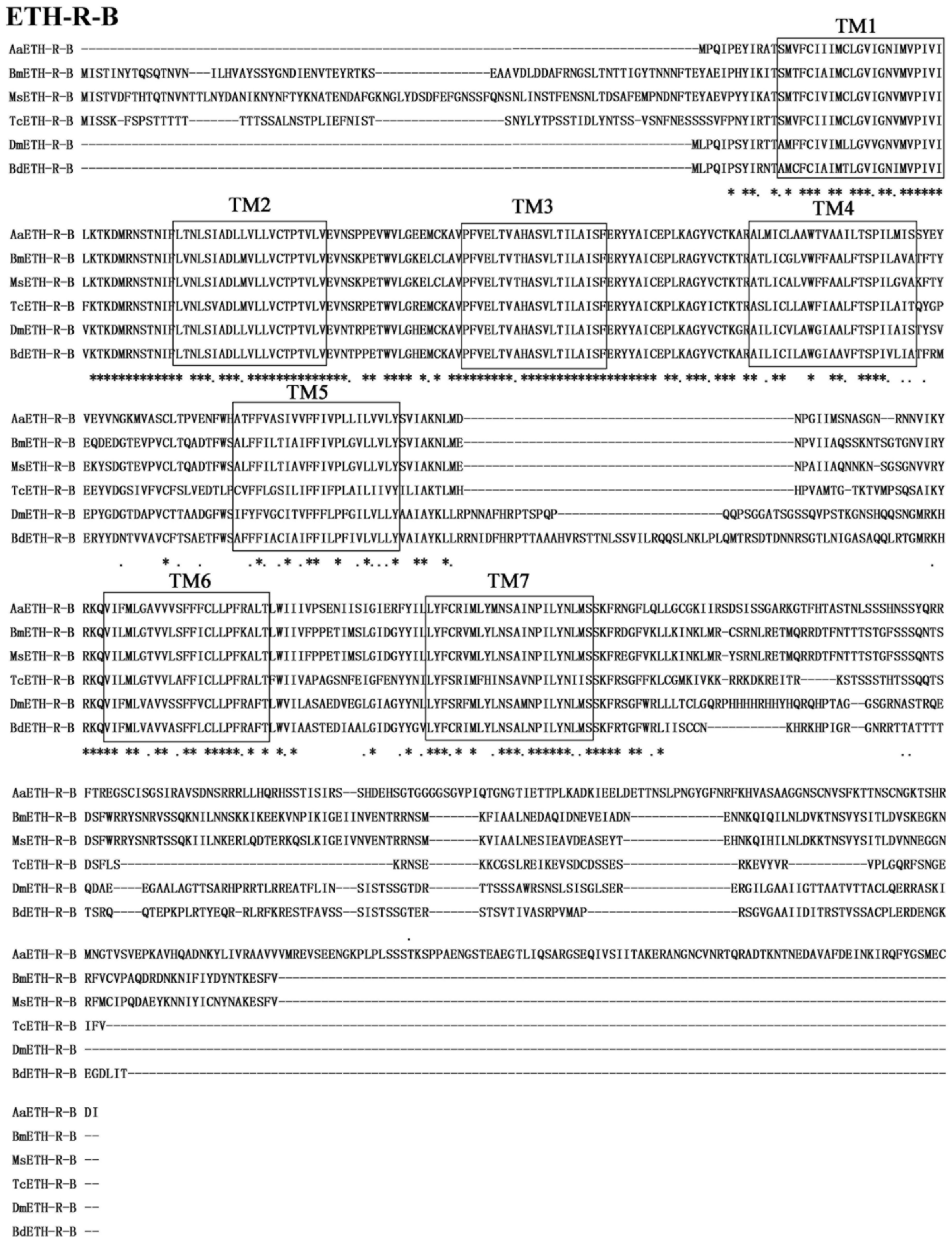

Figure S2. Amino acid sequence alignment of the *BdETH-R-B* with other related GPCRs.

Conservative AA are indicated by asterisks. Seven transmembrane domains are highlighted in black box (TM1-TM7).

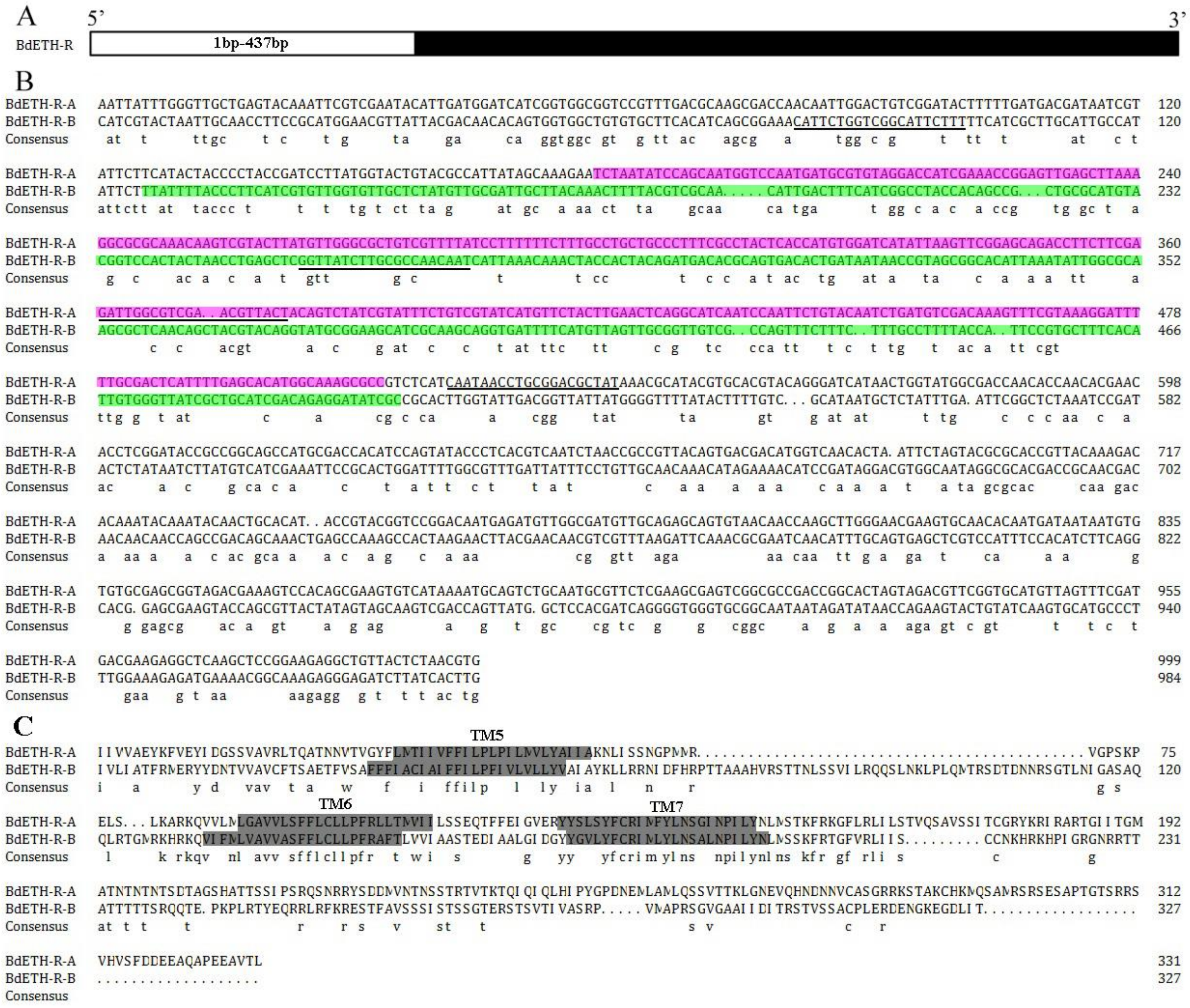

Figure S3. Comparative analysis of alternative splicing exons of *BdETH-R* in *Bactrocera dorsalis*. (A) Schematic diagram of the structure of *BdETH-R* cDNA. Nucleotide positions 1-437 bp was shared by *BdETH-R-A* and *BdETH-R-B*. The black box indicates splice alternatively. ClustalW alignment of nucleotide (B) and deduced amino acid (C) sequences of the alternative exons A and B of *BdETH-R*. Primers for qPCR analysis are underlined. Primers for dsRNA synthesis are highlighted in pink (for *BdETH-R-A*) and green (for *BdETH-R-B*). TM, transmembrane domains. The three transmembrane domains are numbered as TM5-TM7 in alternative splicing exons.

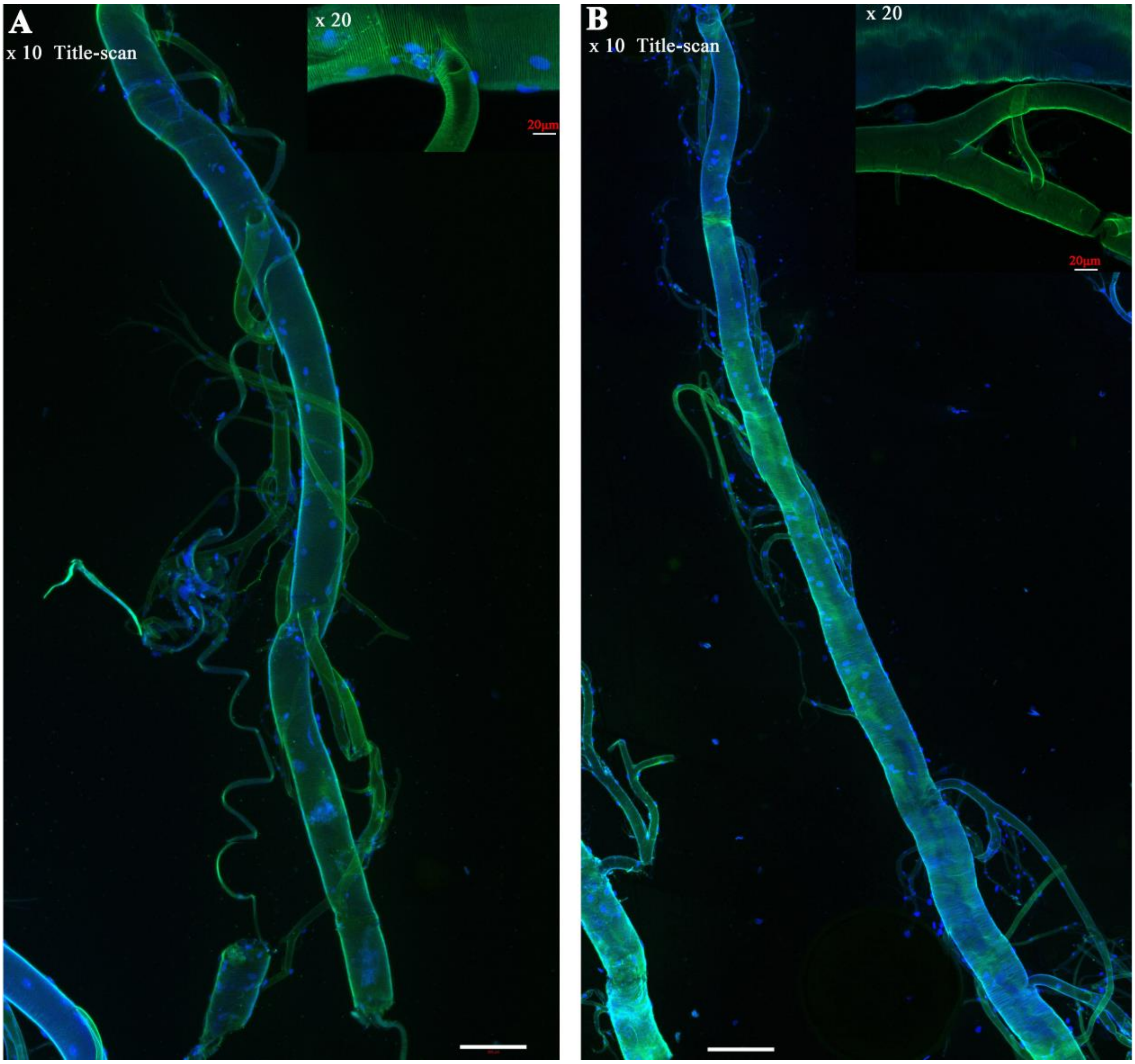

Figure S4. Negative control in the immunohistochemistry and *in situ* hybridization experiments with title-scan method. (A) The negative control for larvae trachea in the immunohistochemistry experiment. (B) The negative control for larvae trachea in the *in situ* hybridization experiment.

Table S1. Primer sequences used in this study

Target	Direction	Sequence 5' to 3'
BdETH (the first-round)	Forward	ACAGTGTATGGGAACAAGCG
	Reverse	TACCATATTCAATGCCCAAT
BdETH (the second-round)	Forward	AGAAAATATGCATTCCTCTTCGCT
	Reverse	ATTCCTAATCTAGTTGATTGTAGC
BdETH-R-A(ORF sequence)	Forward	ATGCTGCCACAGATTCCCTCTTA
	Reverse	TCACGTTAGAGTAACAGCCT
BdETH-R-B(ORF sequence)	Forward	ATGCTGCCACAGATTCCCTCTTA
	Reverse	TCAAGTGATAAGATCTCCCTC
BdETH (qRT-PCR)	Forward	CGTAAACCGGATCAACGACT
	Reverse	GCGCATAAAGTCTCCACCAT
BdETH-R-A (qRT-PCR)	Forward	GATTGGCGTCGAACGTTACT
	Reverse	ATAGCGTCCGCAGGTTATTG
BdETH-R-B (qRT-PCR)	Forward	CATTCTGGTCGGCATTCTTT
	Reverse	ATTGTTGGCGCAAGATAACC
BdETH (dsRNA)	Forward	taatacgactcactatagggCCATTGTCAGGGCAATGAA
	Reverse	taatacgactcactatagggGTTGTAATATATATAATCG
BdETH-R-A (dsRNA)	Forward	taatacgactcactatagggTCTAATATCCAGCAATGGT
	Reverse	taatacgactcactatagggGGCGCTCTGCCACGTACTT
BdETH-R-B (dsRNA)	Forward	taatacgactcactatagggTTATTTTACCCTTCATCGTG
	Reverse	taatacgactcactatagggGCGATATCCTCTGTCGATGCA
GFP (dsRNA)	Forward	taatacgactcactatagggCAGTTCTTGTTGAATTAGATG
	Reverse	taatacgactcactatagggTTTGGTTTGTCTCCCATGATG
Bd-specific probe *ETH*	Forward	ACATTTGGCGTTTTGCTCTT
(*situ* hybridization)	Reverse	CTTCGGCTGGATTCATAAGC
α-Tubulin (internal reference)	Forward	CGCATTCATGGTTGATAACG
	Reverse	GGGCACCAAGTTAGTCTGGA

Table S2. Details of ETH protein and ETH receptors sequences used for analysis.

No.	Species	Gene Name	Accession Numbers
1	*Bactrocera dorsalis*	BdETH	KY705397
2	*Bombyx mori*	BmETH	NP_001165743
3	*Manduca sexta*	MsETH	AAD45613.1
4	*Aedes aegypti*	AaETH	ABI93272.1
5	*Drosophila melanogaster*	DmETH	AAF47275.1
6	*Tribolium castaneum*	TcETH	EFA07492.1
7	*Aedes aegypti*	AaETH-R-A	DQ864500
		AaETH-R-B	DQ864501
8	*Drosophila melanogaster*	DmETH-R-A	NP_650960
		DmETH-R-B	NP_996255
9	*Tribolium castaneum*	TcETH-R-A	ABN79653
		TcETH-R-B	ABN79654
10	*Manduca sexta*	MsETH-R-A	AAX19163
		MsETH-R-B	AAX19164
11	*Bombyx mori*	BmETH-R-A	AB330426
		BmETH-R-B	AB330427
12	*Bactrocera dorsalis*	BdETH-R-A	MF039910
		BdETH-R-B	MF039911
